# Individualized retrograde endoscopic transoccipital-fourth ventricular-midbrain aqueduct to third and lateral ventriculoperitoneal shunt for complex multilocular hydrocephalus with isolated fourth ventricle: a case report

**DOI:** 10.3389/fmed.2025.1411371

**Published:** 2025-05-23

**Authors:** Bodong Wang, Xiaogang Wang, Qiang Liu, Ruwen Ma, Weijie Zhu

**Affiliations:** Department of Neurosurgery, The 960th Hospital of the People’s Liberation Army (General Hospital of Jinan Military Command), Ji’nan, China

**Keywords:** endoscopic surgery, hydrocephalus, individualized therapy, isolated fourth ventricle, retrograde shunt

## Abstract

Hydrocephalus is a condition frequently encountered in neurosurgery. An isolated fourth ventricle represents one of the most challenging forms of hydrocephalus. Currently, there are few clinically mature single-session surgical solutions available for the treatment of complex hydrocephalus with an isolated fourth ventricle that exhibits both obstructive and communicating features. Herein, we report a case of complex hydrocephalus with an isolated fourth ventricle treated with an endoscopic transmesencephalic aqueduct retrograde shunt. The patient recovered well postoperatively, with significant improvement in hydrocephalus symptoms. Based on a thorough analysis of the etiology, we suggest that shunt surgery using an endoscopic transoccipital-fourth ventricle-midbrain aqueduct-third ventricle-lateral ventricle retrograde approach can be applied to patients with complex hydrocephalus, using an individualized plan. The successful treatment in our case provides a reference for the management of patients with complex hydrocephalus and an isolated fourth ventricle.

## 1 Introduction

Hydrocephalus is a common complication encountered in neurosurgery. Unless congenital, it is usually secondary to bleeding or infections. Among these, complex hydrocephalus, where hydrocephalus is combined with an isolated fourth ventricle (IFV), remains challenging (1). Various surgical treatment options have been reported, including lateral ventricular shunt, fourth ventricular shunt, third ventriculostomy, and midbrain aqueductoplasty, with or without stent placement (2, 3). These approaches have different indications and comparative advantages; however, they also have distinct limitations in treating complex hydrocephalus.

**Figure 1:**
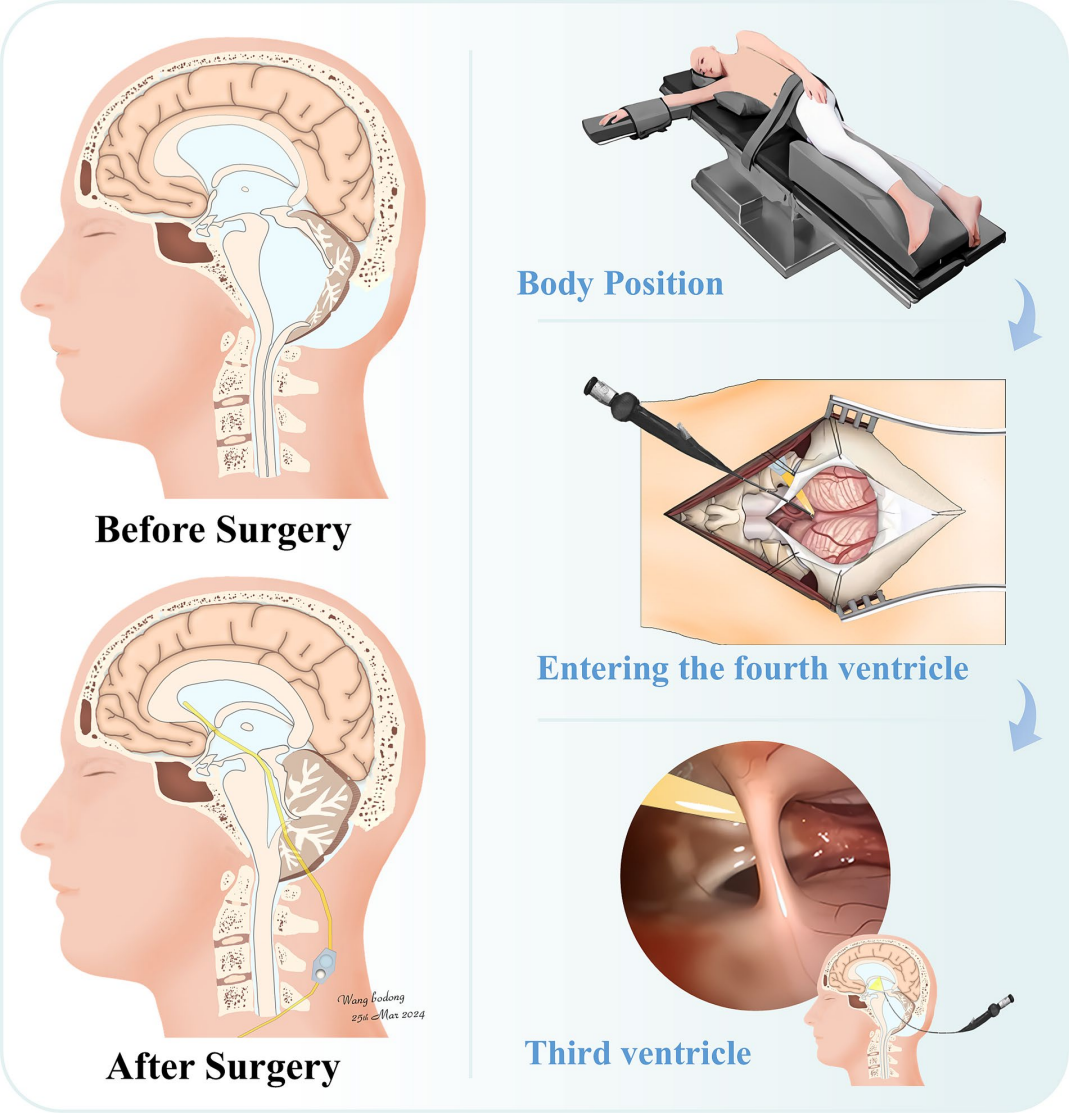
Schematic diagram of the individualized retrograde endoscopic transoccipital-fourth ventricular-midbrain aqueduct to third and lateral ventriculoperitoneal shunt. The left column illustrates the patient’s brain condition before and after surgery. Prior to the operation, the entire ventricular system was dilated, particularly the fourth ventricle. The corpus callosum and midbrain were compressed and thinned, accompanied by suboccipital subcutaneous fluid accumulation. Following the shunt operation, the ventricular system retracted, relieving compression of the corpus callosum and midbrain, and the suboccipital subcutaneous fluid accumulation disappeared. The right column depicts the surgical procedures, including the right lateral decubitus position, the anatomical diagram of the shunt entering the fourth ventricle post-craniotomy, and the endoscopic view of the third ventricle.

With advancements in endoscopic technology, it is highly feasible to place a shunt from the fourth ventricle through the midbrain aqueduct into the lateral ventricle under full visualization (4). Herein, we report a case of complex hydrocephalus with an IFV treated with an endoscopic transmesencephalic aqueduct retrograde shunt, considering the individual case (Figure 1). Postoperatively, the patient experienced marked improvement in symptoms such as headache, dizziness, nausea, vomiting, and urinary incontinence. During the 1-year follow-up, the patient was able to live independently and was satisfied with the surgery’s outcomes.

## 2 Case description

A 52-year-old woman with a history of hypertension presented with a subarachnoid hemorrhage (SAH) due to a posterior inferior cerebellar artery aneurysm, accompanied by diffuse intraventricular hemorrhage. Six months prior, she was urgently admitted to the emergency department following a sudden onset of coma, presenting with elevated blood pressure. Cranial computed tomography (CT) scans revealed diffuse intraventricular and SAH, while CT angiography identified a posterior inferior cerebellar artery aneurysm. At the local hospital, she underwent bilateral ventricular puncture and drainage, followed by hematoma evacuation in the fourth ventricle and aneurysm resection. One month post-treatment, she was discharged with a clear mind and a mild limp in her right lower limb.

Four months later, the patient developed symptoms of headache, nausea, vomiting, anorexia, and movement disorders, attributed to an IFV. She then underwent posterior fossa adhesiolysis and midbrain aqueduct dredging at another hospital. However, her symptoms reappeared a month later, prompting her to seek treatment at our neurosurgery department. She exhibited progressively worsening movement disorders in all limbs and eyes, speech disturbances, and urinary incontinence. Upon admission, the patient presented severe symptoms of midbrain compression, including consciousness disturbances and eye movement disorders. Additionally, she showed signs of high intracranial pressure, such as headaches, nausea, and vomiting. Physical examination revealed a stiff neck, grade III muscle strength in all limbs with hypotonia and hypoesthesia, and an inaccurate finger-nose test. Romberg’s sign and the heel–knee-shin test could not be completed, while other examination results were normal. She was diagnosed with recurrent IFV accompanied by complex hydrocephalus, with features of both communication and obstruction.

**Figure 2:**
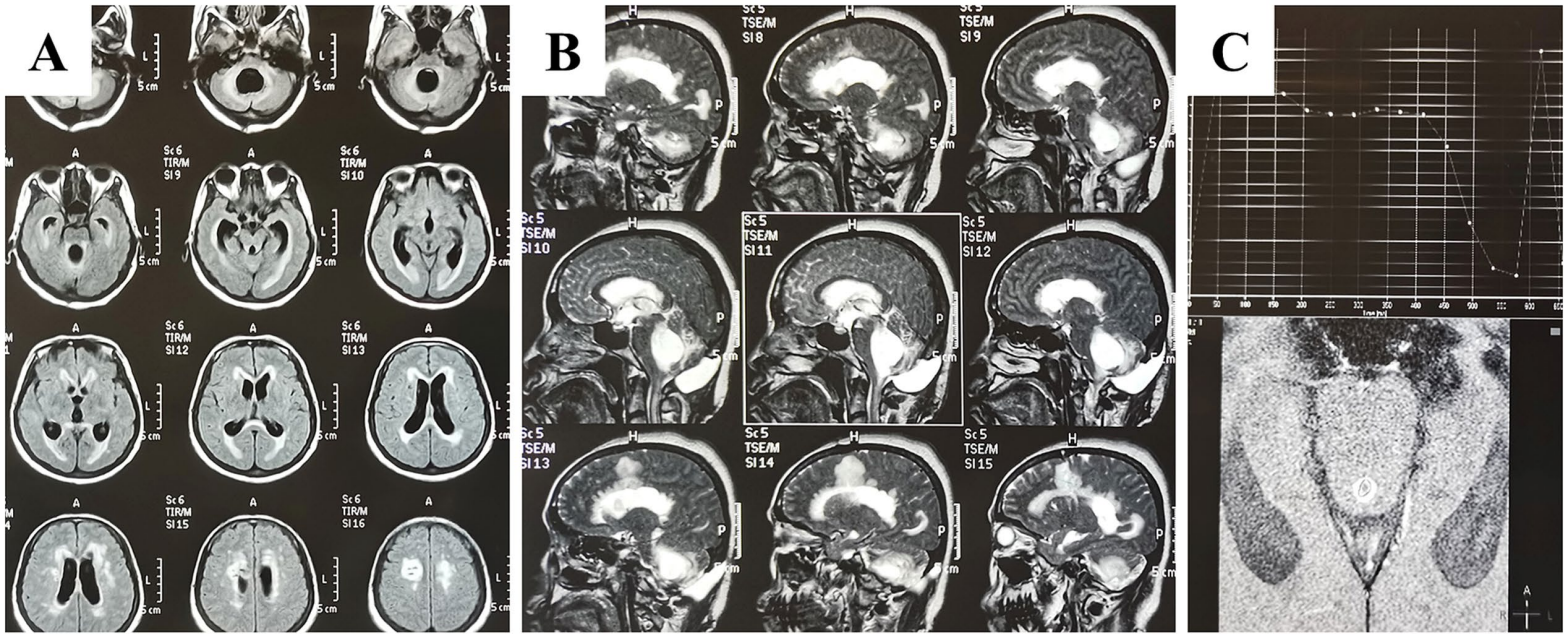
Pre-operative MRI images. **(A)** Axial MRI in T2 Flair scans displaying dilated bilateral cerebral ventricles, third ventricle, midbrain aqueduct, and fourth ventricle, accompanied by interstitial edema. **(B)** Sagittal MRI in T2 phase showing the dilated ventricles and interstitial edema. **(C)** Phase-contrast cine MRI of cerebrospinal fluid demonstrating an obstruction in the midbrain aqueduct. *Abbreviations:* MRI, magnetic resonance imaging; Flair, fluid-attenuated inversion recovery.

A lumbar puncture for the cerebrospinal fluid (CSF) test was not performed to avoid herniation. CT and MRI revealed dilation of the ventricular system, with markedly obvious dilation of the fourth ventricle; interstitial edema; and occipital subcutaneous hydrops (Figures 2A,B). Phase-contrast cine-MRI (PC cine-MRI), which offers a non-invasive, qualitative, and quantitative method for fluid assessment, confirmed the presence of an obstruction in the upper end of midbrain aqueduct (Figure 2C). Her critical condition necessitated surgical intervention. Therefore, we utilized a suboccipital transvelum approach to unblock the adhesion of the upper midbrain aqueduct and third ventricle, combined with retrograde catheterization through the fourth ventricle and the midbrain aqueduct to the third and through the foramina of Monro into the lateral ventricle.

**Figure 3:**
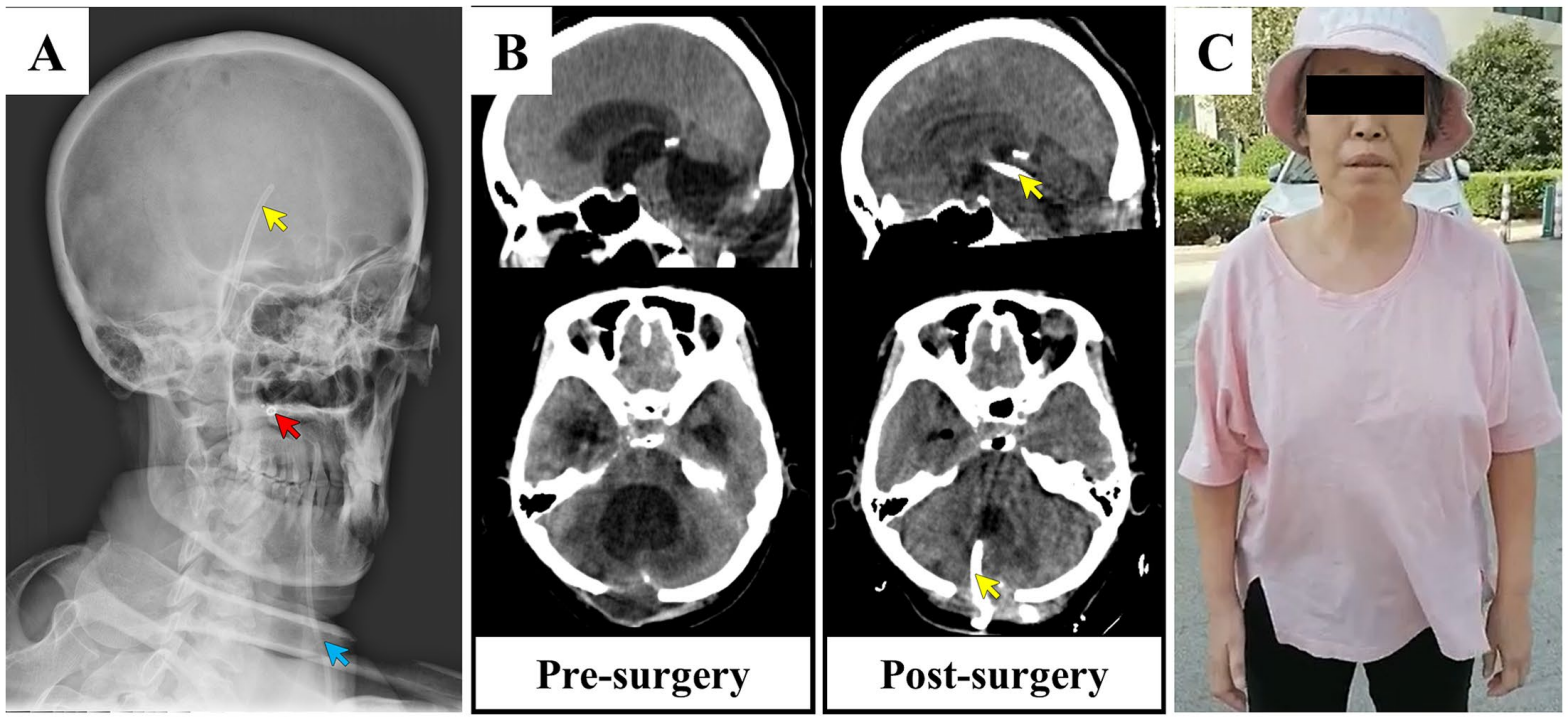
Post-operative images of the patient and CT comparison before and after surgery. **(A)** Postoperative X-ray image revealing the shunt tube located within the ventricle (indicated by the yellow arrow) and the valve positioned beneath the skin (marked by the red arrow). **(B)** Comparative CT images taken before and after surgery, showing alterations in the ventricular system. The postoperative CT scan indicates a reduction in the size of the temporal horn of the third ventricle, the fourth ventricle, and the lateral ventricle. The yellow arrow points to the location of the intraventricular shunt tube. **(C)** Follow-up photographs of the patient taken one year after discharge, providing visual evidence of the patient’s condition and recovery. CT, computed tomography.

During surgery, clear CSF flowed out after a longitudinal skin incision was made through the original surgical incision. This finding reflected the presence of intracranial hypertension. After the partial resection of the cerebellar tonsils, an endoscope was inserted into the fourth ventricle. The fourth ventricle was dilated, and the rear of the brainstem was compressed and deformed. Observing the aqueduct upwards under endoscopy, the lower part of the aqueduct was seen to be expanded, but the upper end was narrowed by adhesions. Then, a 2.7-mm endoscope was inserted to probe upward, and a septum was found in the aqueduct. After removing the septum and adhesions from the aqueduct, the third ventricle became visible. After repeated washing and clearing of the ventricles, a shunt tube was placed into the left lateral ventricle through the fourth ventricle–midbrain aqueduct–third ventricle–interventricular foramen, as the left lateral ventricle was slightly larger than the contralateral side. The end of the shunt tube was fixed to the dura mater, and the shunt valve was buried deep under the occipital skin. The postoperative images (Figure 3A) display the route and position of the shunt tube, while the comparative CT images (Figure 3B) illustrate the alterations in the ventricular system.

The size of the entire ventricular system, including the lateral, third, and fourth ventricles, and symptoms of headache, nausea, vomiting, anorexia, speech disturbance, and urinary incontinence significantly improved after this surgical treatment. During the 1-year follow-up, the patient’s eye and limb movements were normal, and she lived independently (Figure 3C).

## 3 Discussion

A previous intraventricular hemorrhage led to arachnoid malabsorption, causing communicating hydrocephalus. Secondary subependymal gliosis and adhesive arachnoiditis obstructed the aqueduct, as well as Luschka’s and Magendie’s foramina, resulting in obstructive hydrocephalus and an isolated fourth ventricle (IFV). In this case, the patient had a surgical history of midbrain aqueduct dredging, but obstruction recurred postoperatively. The obstruction at the upper end of the midbrain aqueduct was confirmed by phase-contrast cine-MRI (PC cine-MRI). Sakhare et al. reported that cerebrospinal fluid (CSF) flow can be reliably measured at the cerebral aqueduct area using PC-MRI, underscoring its potential as a valuable tool for assessing flow dynamics within the central nervous system (5). Considering that the obstruction was caused by adhesions resulting from inflammatory mediators and gliosis in the ventricular system, we anticipated that re-obstruction might occur after re-unblocking.

The options for hydrocephalus surgery include a lateral ventriculoperitoneal (V-P) shunt, fourth V-P shunt, endoscopic third ventriculostomy (ETV), and midbrain aqueductoplasty, with or without a stent. These surgeries can be performed alone or in combination. Table 1 outlines some indications, advantages, and limitations of existing surgical methods. In this patient, the midbrain aqueduct was partially obstructed and the fourth ventricle was isolated. Therefore, a single lateral or fourth V-P shunt could not resolve the problem. More seriously, a lateral V-P shunt could cause compression from the fourth ventricle to the midbrain, aggravating the disturbance of consciousness, and even endangering her life. Furthermore, the combination of lateral and fourth V-P shunts could cause difficulties in controlling the pressure difference between the supratentorial and subtentorial ventricles.

**Table 1:** Comparison of different surgical options.

Surgical options	Indications	Advantages	Limitations	Complication Rate	Notes
Lateral V-P Shunt	Hydrocephalus	Well-established, widely used.	Pressure difference issues, high complication rate in complex cases.	Moderate	Suitable for general cases
Fourth V-P Shunt	Isolated Fourth Ventricle (IFV)	Directly addresses fourth ventricle, effective infratentorial hydrocephalus treatment options.	High complication rate, pressure control issues.	High	High complication rate in comparison
Endoscopic third ventriculostomy (ETV)	Aqueductal stenosis	Non-shunt option, durable.	Not suitable for all types of hydrocephalus.	Low	Preferred for aqueductal stenosis
Midbrain aqueductoplasty (and stent placement)	Aqueductal stenosis	Re-establishes CSF pathways.	High risk of failure, less effective than fourth V-P shunt.	High	Alternative to ETV
ETV + midbrain aqueductoplasty (and stent placement)	IFV with hydrocephalus	Both surgeries can be performed at the same time.	Requires two surgeries, not suitable for communicating hydrocephalus, high risk of failure, less effective than fourth V-P shunt.	High	High complication rate in comparison
Midbrain aqueductoplasty (and stent placement) + Lateral V-P Shunt	IFV with hydrocephalus	Re-establishes CSF pathways.	Requires two surgeries, high risk of failure.	High	High complication rate in comparison
Lateral V-P Shunt + Fourth V-P Shunt	IFV with hydrocephalus	Both shunts can be performed at the same time.	Requires two shunts, pressure difference control issues.	High	High complication rate in comparison
Endoscopic Transoccipital-Fourth Ventricular-Midbrain Aqueduct to Third and Lateral Ventriculoperitoneal Shunt	IFV with hydrocephalus	One operation, shunt can be used as a stent to keep the cerebral aqueduct open, the fourth and third ventricles are fully visible during the operation.	Narrow indications, risk of midbrain damage.	High	High complication rate in comparison

According to the literature, ETV is the preferred option for the treatment of hydrocephalus combined with midbrain aqueduct obstruction, except in some infratentorial ventricular obstructive cases where ventriculostomy is not suitable. However, ETV is also suitable for some types of isolated fourth ventricle (IFV). In 2020, Wakabayashi et al. reported a case series describing the endoscopic diagnosis and treatment strategy for disproportionately large communicating fourth ventricle (DLCFV), a rare subtype of IFV characterized by dilation of the fourth ventricle, regardless of the size of the lateral ventricles, with no apparent obstruction of the cerebral aqueduct (6). He also conducted a PubMed search, revealing that ETV was performed in all DLCFV patients. Only two patients required additional endoscope-assisted placement of a fourth V-P shunt due to severe adhesion of the interpeduncular cistern from subarachnoid hemorrhage (SAH). Furthermore, ETV is also reported as a viable option for treating fourth ventricular outlet obstruction in patients older than two years of age. In contrast, a V-P shunt is a favorable option for infants younger than six months of age due to the high failure rate of ETV in this age group (7). Lastly, in IFV cases, fourth ventricle-peritoneal shunt surgery is usually employed because ETV alone cannot completely resolve fourth ventricle hydrocephalus; however, the complications of a fourth ventricle–peritoneal shunt are much higher than those of a lateral V-P shunt (2, 6). In our case, ETV would not effectively relieve the IFV.

Midbrain aqueductoplasty has long been considered an alternative to endoscopic third ventriculostomy (ETV) for the treatment of idiopathic aqueductal stenosis-associated hydrocephalus (2, 6). Aqueductoplasty can be performed through the frontal horn of the lateral ventricle or through the suboccipital fourth ventricle and is a recognized method for reestablishing normal cerebrospinal fluid (CSF) pathways and balancing supratentorial and infratentorial pressures (8). Endoscopic aqueductal stent placement (stentoplasty) is the most common anatomical method to resolve an IFV and establish a normal CSF circulation pathway (9). This procedure dates back to the 1920s when Dandy et al. pioneered midbrain aqueduct stent placement by inserting a rubber tube into the midbrain aqueduct. However, this surgery is associated with risks such as postoperative oculomotor nerve palsy and stent migration. Aqueductoplasty today also carries a high risk of failure and is usually less effective than a fourth ventricular shunt in relieving hydrocephalus (2). In our case, there were two options to access the ventricular system endoscopically: through a frontal or a suboccipital transvelum approach. The endoscopic frontal horn approach may have been hindered by the connexus interthalamicus before it entered the aqueduct, while the fourth ventricle could not be clearly observed. Therefore, we opted for the suboccipital transvelum approach to endoscopically remove the adhesion in the upper midbrain aqueduct and third ventricle, and with a retrograde catheter inserted through the fourth ventricle and midbrain aqueduct to the third ventricle, continuing through the foramina of Monro into the lateral ventricle. This approach allowed for better observation of the interior of the third and fourth ventricles, enabling the removal of adhesions. Notably, Gallo et al. described a successful treatment of trapped fourth ventricle by fenestration of the superior medullary velum through an infratentorial approach in a 20-month-old child with a functional supratentorial V-P shunt and an aqueductal anatomy not favorable for stenting (10). However, we recommend that this operation be performed with caution because, unlike the inferior medullary velum, the superior medullary velum has trochlear nerve fibers passing through it.

Additionally, a meta-analysis by Fallah et al. revealed that aqueductoplasty is an effective procedure with moderate morbidity. Factors such as older age at surgery, congenital etiology, and the use of a stent predict a favorable outcome in delaying the need for a second CSF diversion procedure. Moreover, Imperato et al. conducted a retrospective analysis of 33 children with hydrocephalus and isolated fourth ventricle (IFV) treated between 1999 and 2019. All underwent endoscopic aqueductoplasty and/or aqueductal stent placement. While ten patients did not require further surgery, 23 underwent additional procedures. Among these, 13 had a new aqueductoplasty with aqueductal stent placement, and the remaining ten required various other procedures to manage hydrocephalus. Therefore, aqueductoplasty is only recommended for patients in whom endoscopic third ventriculostomy (ETV) is not feasible (11). Furthermore, endoscopic aqueductoplasty requires the implantation of a stent within the midbrain aqueduct to prevent membranous structures from blocking it. However, the issue of impaired CSF absorption in communicating hydrocephalus cannot be fully resolved by existing methods. The patient in our case is an adult, which lowers the relative risk of surgery compared to a child. Besides, the V-P shunt resolved the communicating hydrocephalus, while the shunt tube placed in the midbrain aqueduct functioned as a stent.

In this case, we present an endoscopic transaqueductal shunt surgery for patients with an isolated fourth ventricle (IFV) and communicating hydrocephalus. Depending on individual circumstances, a frontal or suboccipital approach could be used to pass the shunt through the midbrain, placing it in the fourth or lateral ventricle. Based on the treatment process in our case, we propose the following key points for the surgery: choose an endoscope with a smaller diameter whenever possible, and soft endoscopes offer several advantages over hard ones. To ensure that the brainstem is not damaged, carefully select the appropriate angle and field of view, which tests the operator’s skill, and select patients who are suitable for this surgical method. Partial obstruction of the midbrain aqueduct is usually accompanied by the expansion of the aqueduct outlet, facilitating endoscopic entry. However, it must be noted that surgery may cause brainstem damage in patients with complete atresia. Severe cases can lead to respiratory and circulatory failure, coma, and even death. At the least, it may affect the movement of the pupils and eyeballs and descending signals in the pyramidal tract. The success of our case largely relied on careful evaluation of the particularities: the upper end opening of the midbrain aqueduct was obstructed by adhesions, while the middle and lower segments were significantly dilated, providing natural advantages for endoscopic entry. We recommend that a more comprehensive evaluation be performed before this procedure to reduce the risk of damaging the periaqueductal gray matter.

## 4 Conclusion

We have demonstrated in clinical practice that the placement of a retrograde catheter-peritoneal shunt through the fourth ventricle–midbrain aqueduct–third ventricle–lateral ventricle under endoscopy is feasible and offers a promising surgical method for the treatment of complicated hydrocephalus. This technique highlights the importance of advanced endoscopic skills and anatomical knowledge, given the potential risks to the midbrain.

Broader clinical implications include the potential for this method to become a standardized procedure for treating complex multilocular hydrocephalus with isolated fourth ventricle, provided further evidence supports its safety and efficacy. Further clinical research is essential to evaluate the long-term outcomes and effectiveness of this individualized surgical approach.

Additionally, future studies could focus on improving endoscopic equipment and developing standardized operating procedures, enhancing the reproducibility and safety of this technique across diverse clinical settings. By addressing these aspects, we can better understand the potential of this method to improve patient outcomes.

## Data Availability

The original contributions presented in the study are included in the article/supplementary material, further inquiries can be directed to the corresponding authors.
